# Audit logs to enforce document integrity in Skyline and Panorama

**DOI:** 10.1093/bioinformatics/btaa547

**Published:** 2020-05-28

**Authors:** Tobias Rohde, Rita Chupalov, Nicholas Shulman, Vagisha Sharma, Josh Eckels, Brian S Pratt, Michael J MacCoss, Brendan X MacLean

**Affiliations:** b1 Department of Genome Sciences, University of Washington, Seattle, Washington 98195, USA; b2 LabKey, San Diego, CA 92101, USA

## Abstract

**Summary:**

Skyline is a Windows application for targeted mass spectrometry method creation and quantitative data analysis. Like most graphical user interface (GUI) tools, it has a complex user interface with many ways for users to edit their files which makes the task of logging user actions challenging and is the reason why audit logging of every change is not common in GUI tools. We present an object comparison-based approach to audit logging for Skyline that is extensible to other GUI tools. The new audit logging system keeps track of all document modifications made through the GUI or the command line and displays them in an interactive grid. The audit log can also be uploaded and viewed in Panorama, a web repository for Skyline documents that can be configured to only accept documents with a valid audit log, based on embedded hashes to protect log integrity. This makes workflows involving Skyline and Panorama more reproducible.

**Availability and implementation:**

Skyline is freely available at https://skyline.ms.

**Supplementary information:**

Supplementary data are available at *Bioinformatics* online.

## 1 Introduction

In the Skyline software for targeted mass spectrometry (Maclean *et al.*, 2010), users create and work on documents, which contain settings, targets and processed data. With over 10 000 users in dozens of countries, Skyline is increasingly being used in collaboration by researchers. Skyline documents are shared across the world and published as supplementary information through the Panorama Public data repository (Sharma et al., 2014). Prior to the introduction of audit logging, it was nearly impossible to know how a Skyline document and its data were processed without detailed notes kept during creation and processing of the document. This could make reproducing and validating the experimental results challenging. There has also been demand from pharmaceutical companies to use Skyline in regulated environments for purposes such as drug development. Regulated environments have strict requirements for software use such as Title 21 Part 11 of CFR by the FDA, which requires the use of ‘secure, computer-generated, time-stamped audit trails’. These issues motivated the implementation of audit logging in Skyline to track all changes made to a Skyline document. The audit log contains hashes of its contents and the corresponding document to discourage users from modifying the log or making untracked changes to the document. This ensures data integrity and prevents the average user from tampering with the audit log, though it would not prevent a determined adversary from writing code to do so, which will require future work to prevent. Logging is very common in command-line applications because it is easy to implement. One can simply record the commands run by the command-prompt and their output, ideally with a time stamp. Conversely, audit logging is much less common in graphical user interface (GUI) tools, since user actions are more complex and difficult to describe in text and architectural forethought is required to ensure all document changes get logged. The Skyline audit log was designed to make it easy to navigate through the GUI by following entries in the audit log and thereby reproducing the exact state of a document, given only the audit log and the original data. The audit log was not designed to allow for automatic reproduction of a document state. The audit log is displayed to the user in a grid like the Skyline document grid and can be uploaded and viewed in the web database Panorama, which can be configured to reject documents with a missing or invalid log file. Even modifications made through the Skyline command-line interface are tracked in the audit log and can be reviewed in the audit log grid or Panorama.

## 2 Design and implementation

Skyline is written in C# and uses the .NET Framework and Windows Forms. Our audit logging implementation is built into Skyline and therefore written in C#. The audit log is displayed to the user in a grid with customizable columns, which provides the user with an interactive and easy to use view of the audit log entries (Section 2.1). Logging is based on object comparison (Section 2.2). The audit log is stored as a separate human readable, language independent XML file (Section 2.3), like the Skyline document it accompanies, and the two are linked by hash codes to ensure consistency (Section 2.4).

### 2.1 Audit log entry structure and grid

**Fig. 1.**
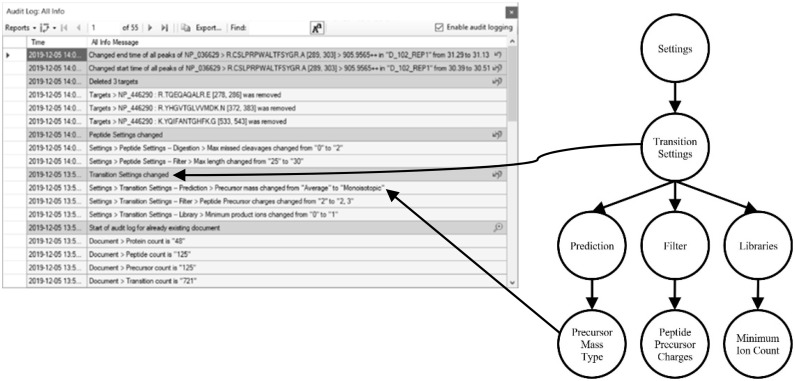
(**a**) The Audit logging grid window displaying several audit log entries with time stamps and log messages. (**b**) The Diff tree generated after modifying several transition settings. A single line summary is constructed by traversing the tree from the root to the first node with multiple children and detailed messages are built by traversing from root to leaf nodes. Two nodes have arrows indicating their corresponding log message in the audit log grid

The Skyline audit log is displayed in a grid based on the Skyline document grid (Fig. 1a). Audit logging is enabled by default but can be disabled through a checkbox in the top right corner of the audit log grid. The basic unit of the audit log is an audit log entry, which represents a single GUI transaction and can consist of multiple property modifications made to the document. For instance, a user can change multiple settings in a single dialog box and all changes applied when the OK button is clicked will be recorded as a single audit log entry with multiple detailed sub-messages representing the individual property changes. In the audit log grid, each line with a dark gray background corresponds to a single audit log entry and user action. If the action cannot be described in a single message, it contains several detail messages shown with a lighter gray background below. Aligned right of the main log messages, are clickable arrows, allowing the user to undo a change and all changes that came after it. However, this is only possible for changes made in the current document session. Audit logging and undo-redo are tightly connected in the Skyline architecture with both being recorded in the same function and sharing change summary text. Some changes have extra information associated with them, such as text when pasting from the clipboard. This extra information can be accessed by clicking on the magnifying glass right of the log message text.

Each audit log entry contains a Time Stamp, an Undo-Redo Message, a Summary Message, a set of All Info Messages, a Username, an optional Reason entered by the user and optional extra information (see Supplementary Material for detailed descriptions of the columns). Since the audit log grid is based on the document grid, it can be customized in the same way as the document grid to show any combination of the above columns using the Reports drop down in the top left corner of the audit log grid window.

### 2.2 Implementation

Skyline uses an immutable tree data structure to model the conceptual document. Every property of a Skyline document is part of this tree. Each time a user performs an action that modifies the document, the *ModifyDocument* method is invoked. The method applies an action in the form of a lambda function that creates new immutable elements, which reference the existing immutable tree. A new root object is created, and it becomes the current document, pushing the previous document root object onto the undo stack. A naïve implementation would be to pass a log message describing the user action as a parameter to *ModifyDocument*. However, this is redundant and difficult to maintain, since each of the several hundred actions in Skyline would require their own hardcoded log messages. Instead, we compare the old and the new documents at the property level. Each feature in Skyline that is tracked by the audit log has a backing object in the document tree and the object structure resembles the structure of the feature as it is presented in the GUI. The audit logging system uses C# reflection to retrieve properties from the old and new objects and compare them. If differences are found, the same algorithm is recursively invoked on the sub properties, until it finds the root of the change. During this recursive traversal, a ‘diff tree’ is created that encodes the differences between the two documents and is later used to create log messages. The immutable tree allows whole branches to be ignored when a parent node is found to be a reference equal to the previous node. While reflection is an expensive operation, the implementation is efficient and has no noticeable impact on performance. Figure 1b is an example of the diff tree generated after changing various transition settings. The tree is used to construct a single line description of the change and a set of detailed descriptions. The log messages generated from this tree are shown in part of Figure 1a. For more details on the nodes see Supplementary Material.

### 2.3 Storage and XML format

The audit log is stored in its own file as language independent, human readable XML and linked to the document through a hash of the document. The audit log does not serialize objects to disk. Instead, it stores textual descriptions of the changes made to those objects. The audit log can be seamlessly transferred between different systems. The time stamp is stored in standard ISO 8601 format, comprised the local time and an offset to UTC, which is automatically converted to the local time zone of the running Skyline instance. The log messages do not contain any language specific information, but rather language invariant identifiers, which are used to look up localized strings at runtime. This allows Skyline to display the audit log in different languages (currently English, Chinese and Japanese). The audit log also contains a fully expanded English version of each log message to simplify integration into Panorama and to make the XML more human readable. For a detailed description of the XML format see Supplementary Material.

### 2.4 Hashing

All hashes in the audit log are SHA-1 hashes. The audit log contains a hash of the document, which is used by Skyline and Panorama to establish correspondence between the Skyline document and the audit log file that is stored separately from the document. The audit log also contains a root hash of all audit log entry hashes. This hash is used to discourage modification of the audit log outside of Skyline and detect corruption of the audit log. Finally, each entry has its own hash, allowing Skyline to pinpoint an exact entry that was modified if someone tries to edit the text outside Skyline. Panorama also makes use of the hashes to validate the audit log and can be configured to reject modified audit logs. For a description of how hashes are created avoiding negative performance impact and how the audit log is validated in Skyline and Panorama, see Supplementary Material.

## 3 Summary and future direction

With the addition of audit logging to Skyline and Panorama, users will be able to collaborate more efficiently by having a detailed record of how a document was processed. Reproducibility of published results will be greatly improved, since the audit log was designed for users to be able to reach the same state of a document given only the original data and the audit log. Our use of hashing improves data integrity and protects against tampering from the average user. The implementation of audit logging brings Skyline closer to complying with requirements for usage in regulated environments and in the future more work will be devoted to achieving this goal. Having a complete audit logging system in a GUI tool is novel and far less common than audit logging for command-line tools. The approach described in this paper is flexible, easy to maintain and applicable to other existing GUI tools. In the future, we expect to use encryption and electronic signatures to enforce document and audit log integrity more strictly in both Skyline and Panorama and to fully protect the audit log from adversaries.

## Supplementary Material

btaa547_Supplementary_Material

## References

[btaa547-B1] MacLean B. et al (2010) Skyline: an open source document editor for creating and analyzing targeted proteomics experiments. Bioinformatics, 26, 966–968. 20147306 10.1093/bioinformatics/btq054 PMC2844992

[btaa547-B2] Sharma V. et al (2014) Panorama: a targeted proteomics knowledge base. J. Proteome Res., 13, 4205–4210. 25102069 10.1021/pr5006636 PMC4156235

