# Supplementary material for: Audit logs to enforce document integrity in Skyline and Panorama
Source: Bioinformatics. 2020 May 28;36(15):4366–8. doi: 10.1093/bioinformatics/btaa547 (PMC7520049; doi:10.1093/bioinformatics/btaa547)
Supplement: btaa547_Supplementary_Material [file btaa547_supplementary_material.docx]

**Audit Logs to enforce document integrity in Skyline & Panorama**

Tobias Rohde, Rita Chupalov, Nicholas Shulman, Vagisha Sharma, Josh Eckels, Brian S Pratt, Michael J MacCoss & Brendan X MacLean

| Columns In the audit log | Page 2 |
| --- | --- |
| Audit log implementation details | Pages 2-4 |
| XML Format | Pages 4-5 |
| Hashing | Page 5 |
| Skyline Audit logging Tutorial | Pages 6-26 |

**Columns in the audit log**

The audit log contains the following columns:

- **Time Stamp:** Time at which the change was made.
- **Undo Redo Message:** The most specific single line message describing the entire document change. This message will also be displayed in Skyline’s toolbar when clicking on the arrows next to the Undo-Redo arrows.
- **Summary Message:** Similar (and often the same) as the Undo Redo Message, but for certain messages shorter.
- **All Info Message(s):** A list of messages that describe the document change in detail.
- **User:** Identity of the user who made the change as authenticated by the local operating system.
- **Reason:** The reason for the change, which can be set by editing the cell in the audit log grid after the change was made. (Optional)
- **Detailed Reason:** A reason that can be set for each of the detailed “All Info messages”. (Optional)
- **Extra Info:** additional information, usually large amount of data pasted into the document. (Optional)

**Audit log implementation details**

Each time the user performs an action that modifies the document, the *ModifyDocument* method is invoked. Below is an example of what a call to *ModifyDocument* method looked like before the implementation of audit logging. The first parameter is a very broad description of the action that is shown in the undo-redo list and the second parameter is a lambda function that accepts a document, makes modifications to one or more copies of nodes of the document tree and returns a new document root.

ModifyDocument("Changed settings", doc => doc.ChangeSettings(newSettings, monitor));

All settings changes would invoke *ModifyDocument* as above, which means that all settings changes would be described with “Changed Settings”, making the implementation of accurate audit logging with the already existing change descriptions impossible. With our object comparison based implementation of audit logging, the *ModifyDocument* method now takes an additional lambda function as a parameter, which accepts an old and new document and uses the documents to generate an *AuditLogEntry* describing the change made by the user. Below is the new invocation of *ModifyDocument*:

ModifyDocument("Changed settings", doc => doc.ChangeSettings(newSettings, monitor),

    AuditLogEntry.SettingsLogFunction);

ModifyDocument still contains the old description, which is used as a backup if generating the audit log entry fails. *AuditLogEntry.SettingsLogFunction* is the main audit logging function which invokes the core audit logging algorithm to recursively compare all properties of the document as described in the paper. It covers most user actions, since most can be described by a list of changes to document properties. Below is an example of a feature in Skyline and its backing object in the document tree


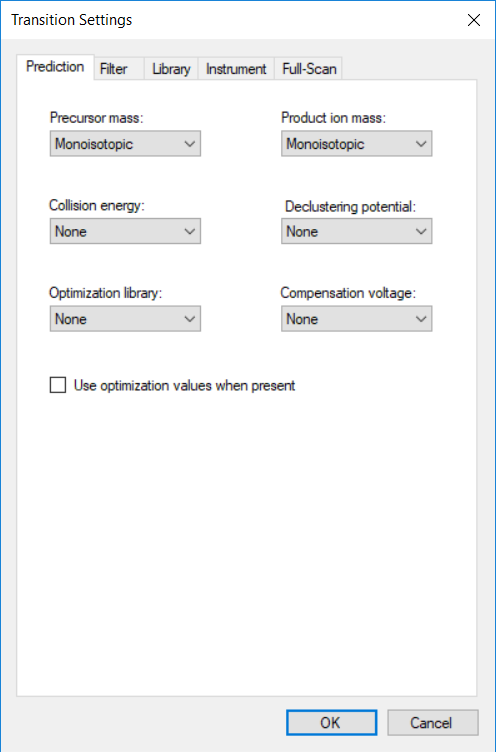


[Track]

public MassType PrecursorMassType;

[Track]

public MassType FragmentMassType;

[TrackChildren]

public CollisionEnergyRegression CollisionEnergy;

[TrackChildren]

public DeclusteringPotentialRegression DeclusteringPotential;

[TrackChildren]

public CompensationVoltageParameters CompensationVoltage;

[TrackChildren]

public OptimizationLibrary OptimizedLibrary;

[Track]

public OptimizedMethodType OptimizedMethodType;

[Track]

public bool UseOptimizationValues;

The code above was slightly modified to fit here. Each dropdown list and the checkbox in the dialog correspond to a property in the object on the right and each of the properties is annotated with an attribute indicating that it should be tracked by the audit log. The **Track** attribute indicates that the audit log should not further recurse into the properties of that object but simply use the property for comparison. The **TrackChildren** attribute on the other hand indicates that the audit log should only compare the property by reference and recurse into the objects properties if the new reference differs from the old reference. The attributes add few lines of code, do not affect the readability of the code and even have the benefit of making it clear to the developer which properties are displayed in the GUI.

One special case of using the track attributes are dialogs where the user configures a process that modifies the document, rather than directly modifying the document. An example of this is importing raw files into the document, where the user goes through a wizard selecting raw files to import and specifying how replicates should be created and named. In this case the audit log should describe the process of the user importing raw files, rather than just the fact that there are new replicates in the document. Just like each feature in Skyline has a backing object in the document tree, each dialog that configures a document modifying process has a backing object which contains all the settings of the process. Thus, when the user confirms such a process, the object comparison algorithm is run on the backing objects of the dialog windows. Since there is no old object for the dialog, a default object containing the default dialog settings is used as the old object.

There are some user actions that are more specialized and cannot easily be described using object comparison, in which case a custom method can be written and passed into *ModifyDocument*.

When the audit log traverses the document tree and finds modifications, “diff nodes” are created which contain several important pieces of information required to construct the audit log entry. Since each node represents a property in the document tree, it contains an object describing the corresponding property. This object contains information about the type of the property and it stores the track attribute, allowing the audit log to retrieve parameters passed to the track attribute. For instance, Prediction, Filter and Libraries are all tabs in the GUI and thus are preceded by “--" in the log message instead of “>”, which is used for all other GUI elements. This and some other information on how to describe properties in text are part of the track attribute. An example of this is below:

[TrackChildren(isTab: true)]

public PeptidePrediction Prediction { get; private set; }

The *isTab* option indicates to the audit log that this property should be preceded by “--". The format using greater than signs and dashes has been used by the Skyline developer team on the support board to describe where settings are located in the GUI. Therefore, it is familiar to many users and has been adopted in the audit log. Diff nodes also contain a property path, which is an ordered list of the property names of parent nodes. The property path is created during the recursive descent down the document tree. This is required to generate a log message for any node, without having to make the diff tree a doubly linked list and traversing it upwards. Furthermore, nodes contain the old object and the new object of the property it represents, which is used for describing the property values in text. Finally, each node contains a list of child nodes and can thus be considered as a tree on its own.

**XML format**

The XML of the audit log resembles its structure in the document, containing a list of XML encoded audit log entries. Below is the XML of an example audit log generated after changing two settings in the Transition Settings:

<?xml version="1.0" encoding="utf-8"?>

<audit_log_root format_version="19.12">

<document_hash>F1VfG8np66mdLBzCWlBxlw9Bxzo=</document_hash>

<root_hash>XBRppBTVnRrG4kNTqKPNzXG1qKY=</root_hash>

<audit_log>

<audit_log_entry skyline_version="19.1.1.309-1dc16c97a (64-Bit)" time_stamp="2019-11-13T01:57:09-

08:00" user="USER-PC\user">

<reason>Main Reason</reason>

<undo_redo>

<type>changed</type>

<name>{0:SrmSettings_TransitionSettings}</name>

<en_expanded>Transition Settings changed</en_expanded>

</undo_redo>

<summary>

<type>changed</type>

<name>{0:Settings}{2:PropertySeparator}{0:SrmSettings_TransitionSettings}</name>

<en_expanded>Settings &gt; Transition Settings changed</en_expanded>

</summary>

<all_info>

<type>changed_from_to</type>

<name>{0:Settings}{2:PropertySeparator}{0:SrmSettings_TransitionSettings}{2:TabSeparator}

{0:TransitionSettings_Prediction}{2:PropertySeparator}

{0:TransitionPrediction_PrecursorMassType}</name>

<name>"{6:MassType_Monoisotopic}"</name>

<name>"{6:MassType_Average}"</name>

<reason>Detailed Reason</reason>

<en_expanded>Settings &gt; Transition Settings -- Prediction &gt; Precursor mass changed from

"Monoisotopic" to "Average"</en_expanded>

</all_info>

<all_info>

<type>changed_from_to</type>

<name>{0:Settings}{2:PropertySeparator}{0:SrmSettings_TransitionSettings}{2:TabSeparator}

{0:TransitionSettings_Filter}{2:PropertySeparator}

{0:TransitionFilter_PeptidePrecursorChargesString}</name>

<name>"2, 3"</name>

<name>"2"</name>

<en_expanded>Settings &gt; Transition Settings -- Filter &gt; Peptide Precursor charges

changed from "2, 3" to "2"</en_expanded>

</all_info>

<hash>TP9aPly2a2p5otJnPnhf9tjq6t0=</hash>

</audit_log_entry>

</audit_log>

</audit_log_root>

First, the audit log contains two hashes, which are described in the next section. They are followed by a list of audit log entries. Each audit log entry element has attributes indicating the skyline version it originated from, the time it was created and the user who performed the action that created it. The example contains a single audit log entry, which was created in version “19.1.1.309-1dc16c97a (64-Bit)” on 11/13/19 at 01:57:09 am by user “USER-PC\user”.

The next element is an optional reason for the entire audit log entry, in this case it is “Main Reason”. The next element is the optional extra info (nonexistent in the example). The next two elements are the undo-redo and summary messages, respectively. The all info messages follow afterwards. In this case there are two all info messages. Afterwards follows an optional English translation of the extra info, which is used by Panorama. This element is only present if there is extra info and it contains tokens. The last element in each audit log entry is a hash of the entire audit log entry. All log messages have the same format. They contain a type element, which indicates what type of action was performed. For instance, the first all info message has type “changed_from_to”. Internally, the type represents a format string that expects parameters. If Skyline is used with English locale, the “changed_from_to” type translates to the string “x changed from y to z” and thus requires three parameters. The parameters are the “name” elements and follow right after in the XML.

In the case of the first all info message the three parameters are

1. “{0:Settings}{2:PropertySeparator}{0:SrmSettings_TransitionSettings}{2:TabSeparator}

{0:TransitionSettings_Filter}{2:PropertySeparator}{0:TransitionFilter_PeptidePrecursorChargesString}”

1. “2, 3”
2. “2”

The type string is formatted with the above parameters, resulting in the following string:

*{0:Settings}…{0:TransitionFilter_PeptidePrecursorChargesString} changed from “2,3” to “2”*.

The name attributes use a special format of the form “{i:s}”, where “s" is a string to be parsed and “i" is an identifier that tells Skyline how to interpret “s”. For instance, in “{0:SrmSettings_TransitionSettings}”, “SrmSettings_TransitionSettings” is a resource string that depending on the current language in Skyline is parsed as “Transition Settings”, if the current language is set to English. The index “0” indicates that this is a simple resource lookup by name. Each token in a log message is parsed in this way and the above log message would be translated as:

*Settings > Transition Settings – Filter > Peptide Precursor charges changed from "2, 3" to "2"*

The fact that we never store any language specific information but rather language invariant identifiers used to lookup localized strings allow Skyline to display audit log messages in different languages, independent of the language of the Skyline instance the audit log was recorded on. The next element is an optional reason provided by the user. This reason is only used by the all info messages. In the first all info message the reason is “Detailed Reason”. Lastly each message contains the entire log message as English text, which is used by Panorama.

The schema for the Audit log XML can be found at:

<https://github.com/ProteoWizard/pwiz/tree/master/pwiz_tools/Skyline/TestUtil/Schemas/AuditLog>

**Hashing**

The audit log contains a SHA1 hash of the document, which is necessary to establish document to audit log correspondence, since the audit log is stored separately from the document. Calculating the hash of the entire document is by far the most expensive hashing operation introduced by the audit log, thus is required an efficient implementation. Skyline uses an XML stream writer for saving the document. This allows us to access individual XML elements as they are being written to the Skyline document file. SHA1 can be computed block-wise and does not require the full text to be hashed at once. Thus, we can compute the hash during document saving by feeding the XML elements from the XML stream into the SHA1 block hashing function. The hash is finalized once the Skyline document is fully written and it is stored for later usage in the audit log. This makes for an efficient implementation and the impact on performance is negligible. Since the document hash is always written to the audit log when the document is saved, it should always match the document under normal circumstances. When Skyline loads the audit log, it reads the hash and compares it to the hash of the document itself. If they match, the audit log is accepted and loaded. If they do not match, it indicates that either the audit log does not belong to the document or that the document was modified without the audit log being aware of it. In both cases, the audit log is rejected as there is no guarantee that the state of the document is fully described by the audit log.

The audit log also contains a hash for each audit log entry and a root hash of all audit log entry hashes. The root hash is used to efficiently ensure that the audit log was not modified outside of Skyline without having to compare each individual audit log entry hash. When the audit log is parsed, the hash for each audit log entry is calculated by hashing the document type, skyline version, timestamp, user, extra info, reason and the English translations of all log messages. The final root hash is compared to the hash calculated at runtime. If they do not match, the audit log is rejected. Since we have access to the actual and expected hashes of each entry, we can use the entry hashes to determine which audit log entries were modified outside of Skyline and show them to the user. For any given audit log entry, the hash is only computed once and cached for any further usage. This is possible since just like the Skyline document itself, audit log entries are immutable. Computing hashes for audit log entries is comparatively inexpensive when compared to hashing the document and thus also has no noticeable performance impact. As noted in the manuscript, the primary purpose of hashing is to improve data integrity and prevent accidental modification of the audit log or the document. While our implementation of hashing prevents the average user form tampering with the audit log, more advanced adversaries could bypass the current hashing mechanism.

**Skyline Audit Logging Tutorial**

Check (<https://skyline.ms/tutorial_audit_log.url>) for the most up-to-date version of this documentation. Periodic revisions of the documentation will occur as additional features are added to Skyline.

Skyline Audit Logging

# Introduction

This tutorial covers how to use the Skyline audit log. The audit logging system keeps track of all document modifications and displays them in an interactive grid, similar to the Document Grid. It benefits from all the features of the Document Grid, such as creating report templates, sorting by columns and editing cells, for example for adding a Reason to a log entry. One of the design goals of the audit log was to give anyone the ability to reconstruct the state of a document, given only the audit log and the original data. This makes it an invaluable tool for a researcher using Skyline. As you will see, the audit log also makes the “Undo-Redo” feature more usable by providing more specific change messages and allowing you to undo changes in the audit log grid itself. To demonstrate important benefits of the audit log, this tutorial is based on the Absolute Quantification Tutorial, in which the absolute abundance of a target peptide is determined using Selected Reaction Monitoring (SRM) mass spectrometry by creating an external calibration curve with an internal standard heavy labeled reference peptide. This tutorial will be mostly focused on how to configure the audit log, how your actions are logged and how to read and work with the audit log. If you find you want to learn more about absolute quantification, you should refer to the Absolute Quantification Tutorial directly, which covers the topic in more detail.

# Getting Started

To start this tutorial, download the following ZIP file:

<https://skyline.ms/tutorials/AuditLog.zip>

Extract the files in it to a folder on your computer, like:

C:\Users\tobiasr\Documents

This will create a new folder:

C:\Users\tobiasr\Documents\AuditLog

The folder should contain a total of 9 RAW files, which you will import into Skyline later.

**Note:** In configuring Skyline for this tutorial, be sure to follow the steps below in the order presented to avoid creating audit logs of these initial setup steps.

- Start Skyline.
- If you are using Skyline for the first time you will be prompted to select a default user interface. In the right-hand corner, ensure that the protein icon
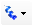
 is showing, indicating that Skyline is presenting the **Proteomics interface**. This can easily be changed in the future.
- On the **Start Page,** click **Blank Document** which looks like this:


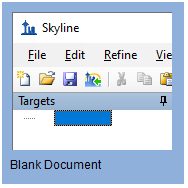


- On the **Settings** menu, click **Default.**
- Click **No** on the form asking if you want to save the current settings.

The document settings in this instance of Skyline have now been reset to the default, but you may still have an audit log entry describing what it took to get from any previous settings back to the defaults.

- On the **File** menu, click **New**.
- When prompted whether you want to save your changes, click the **No** button.

## Opening the audit log

Now open the audit log by doing the following:

- On the **View** menu, choose **Other Grids** and click **Audit Log** (Alt + 4)

This should bring up the **Audit Log** view in its default configuration as shown below:


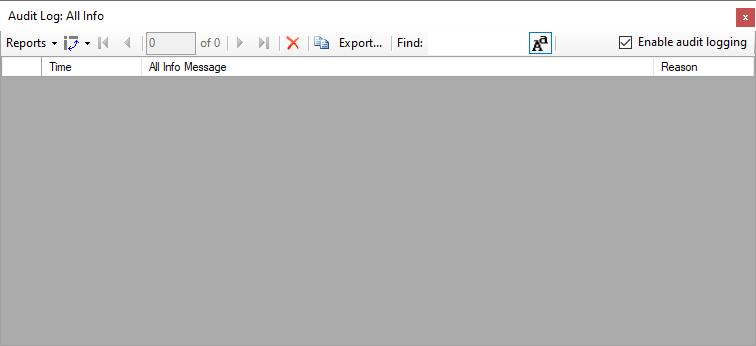


In the top right corner, you can see that audit logging is currently enabled, which is the default. By unchecking the box, audit logging can be disabled. Initially the audit log is showing its default column configuration, displaying the **Time**, **All Info Message**, and **Reason** columns. These columns will be explained in more detail and you will learn how to customize the columns in this grid. Keep this window open throughout the tutorial to observe how log messages appear as you interact with Skyline.

# Configuring Settings for Inserting a New Peptide

Before you insert a new peptide into the document, you need to configure the transition and peptide settings for this experiment as follows:

- On the **Settings** menu, click **Transition Settings**.
- Click the **Filter** tab.
- In the **Product ion selection** box, in the **From** dropdown list select **ion 3** and in the **To** dropdown list ‘**last ion – 1**’.
- Click the **OK** button.

After you click **OK**, several messages will appear in the audit log window. Before taking a closer look at the audit log, first configure the peptide settings as follows:

- On the **Settings** menu, click **Peptide Settings**.
- Click the **Modifications** tab.
- Click the **Edit list** button next to the **Isotope modifications** list.
- Click the **Add** button.
- From the **Name** dropdown list choose ‘Label:13C(6)15N(2) (C-term K)’.
- Click the **OK** button.
- Click the **OK** button in the **Edit Isotope Modifications** form.
- In the **Isotope modifications** list, check the new ‘Label:13C(6)15N(2) (C-term K)’ modification.
- Click the **OK** button in the **Peptide Settings** form.

# Reading the Audit Log

Now the **Audit Log** view should look like this:


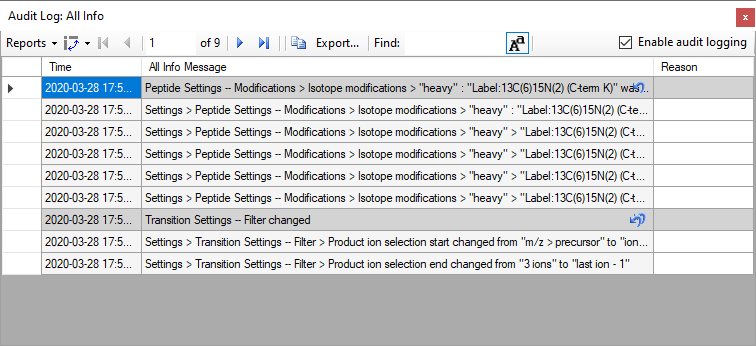


Each dark gray message and all messages below it until the next dark gray message or the end of the audit log represent a single undoable operation. The transition settings and the peptide settings were changed, which creates a total of two audit log entries. By default, new messages appear at the top like an email inbox. Looking at the first entry when the transition settings were changed, the gray message is a one line summary of the entire entry: **Transition Settings – Filter changed**. This message is called the **Undo-Redo message**, because it is also used in the Skyline Undo and Redo dropdown lists. To see this, return to the main Skyline window where you can:

- In the main toolbar, click the small black dropdown arrowhead next to the **Undo** button.

You will see a list of the changes you have made to the Skyline document like this:


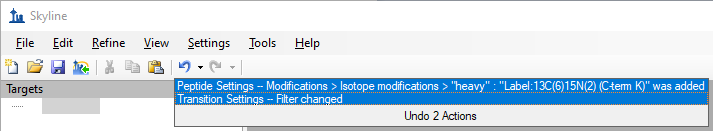


Before audit logging was introduced into Skyline, both messages here would have only said **Changed settings**. Therefore, even if you have audit logging disabled, you can still benefit from it through improved Undo and Redo descriptions.

Return to the **Audit Log** view. The next five messages under the **Undo-Redo message** are the **All Info messages**, which describe every change in detail. Log messages will tell you exactly where a setting changed. For instance, the first message reads **Settings > Transition Settings – Filter > Product ion selection start changed from “m/z > precursor” to “ion 3”**. Recall that this is exactly where we navigated earlier to select the collision energy. The “greater than” symbols indicate that a menu item, while the “--" indicates that what follows is a tab, such as the **Filter** tab. Next look at the message above, which describes the changes you made to the peptide settings. Note that the **Undo-Redo message** is a concise description of the isotope modification you added. Looking below at the **All Info messages**, you can see that the audit log contains the exact definition of the modification you added, despite you not having configured it manually. This is to allow others to reproduce those changes in the future, even if they do not have this particular modification in their Skyline instance, in which case they would have to create it manually.

# Undoing Changes Using the Audit Log View

Again, looking at the **Audit Log** view, you should see that there is a single leftward curving arrow next to the audit log entry at the top that describes the peptides settings changes. This is the same arrow as on the **Undo** button in the toolbar.

- Click the arrow in the top row of the **Audit Log** grid.

This should make the audit log entry disappear and undo the change.

In the main Skyline toolbar and you should see the **Redo** button that was previously a grey arrow pointing rightward, turn blue. To see the dropdown list of changes you can redo:

- Click on the small black arrowhead to the right the **Redo** button in the main toolbar.

You will see the list shown below.


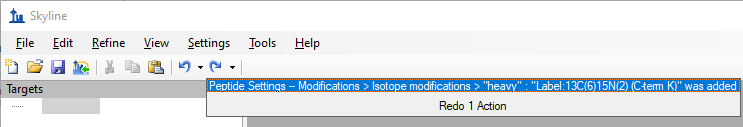


You can see the change you just undid through the **Audit Log** grid.

- Click on the change to redo the operation.

Now return to the **Audit Log** grid and you will see that the audit log entry is back. Note that next to the other audit log entry below it is an icon with two undo arrows. This indicates that if you undo this change, all changes made after this one will also be undone. In this case undoing the transition settings change entry will also undo the peptide settings change entry. This exactly how undo works when using the undo dropdown list in the toolbar. You can undo back to a certain point in the document history, but you cannot revert a single change in the middle of the list. Note that once you close Skyline and open the document, those undo arrows will disappear from the audit log, since only changes made during your current Skyline session can be undone or redone, again consistent with the **Undo** and **Redo** buttons and menu items.

# Inserting a Peptide Sequence

To add a peptide target to your new document perform the following steps:

- On the **Edit** menu, choose **Insert** and click **Peptides**.
- Copy “IEAIPQIDK” to the clipboard and paste it into the first **Peptide Sequence** cell.
- Copy “GST-tag” to the clipboard and paste it into the first **Protein Name** cell.

The form should now look like this:


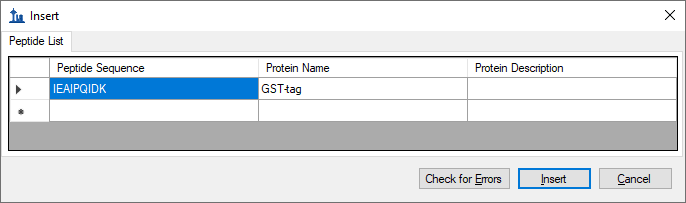


- Click the **Insert** button.

The **Targets** view should show the newly added peptide with a light and a heavy labeled precursor:


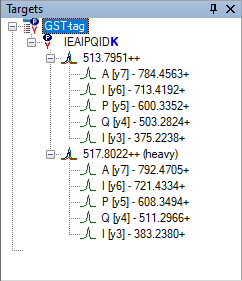


If you do not see all of the elements shown above, do the following:

- On the **Edit** menu, choose **Expand All** and click **Precursors**.

Refer to the **Audit Log** grid and you should see a new message as the first row indicating that you inserted the peptide IEAIPQIDK. There are no other detailed messages about this change. The audit log entry consists of a single audit log messages as shown below:


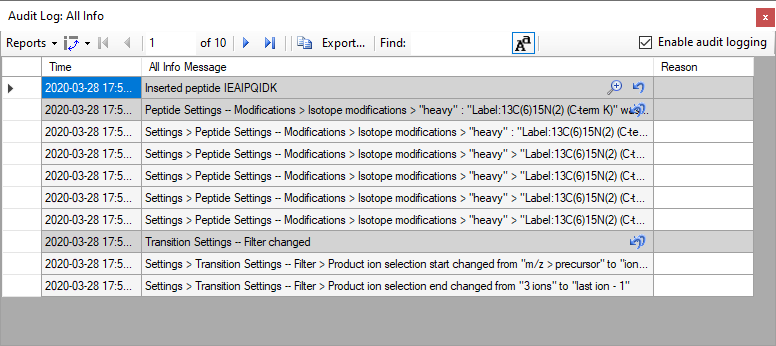


Note that there is a magnifying glass icon next to the new audit log entry. This appears whenever there is extra information associated with an audit log entry.

- Click on the magnifying glass.

This will bring the **Audit Log Extra Information** form as shown below:


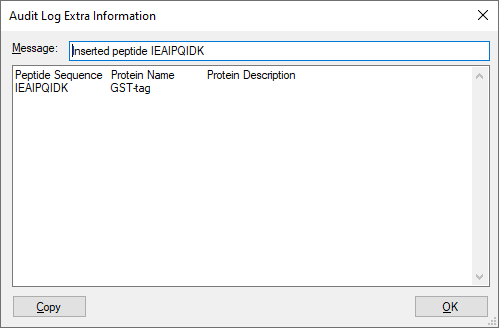


The extra information window shows all the information that was pasted into the grid when inserting the peptide, including the column header names. The cell values are separated by tab characters, which means you can copy entire rows from this window and paste them into the **Insert Peptides** form you just used to reproduce this operation.

- Click the **OK** button to close the **Audit Log Extra Information** form.

Before continuing, you should save the document.

- On the **File** menu in Skyline, click **Save As**.
- Navigate to the AuditLog folder created earlier.
- In the File name field, enter “AuditLogTutorial”.
- Click the **Save** button.
- On the **File** menu, click **Open containing folder**.

In the Windows File Explorer, you should see a file called “AuditLogTutorial.skyl” (along with AuditLogTutorial.sky and .sky.view). If you do not see these file extensions, you may need to show file extensions in the File Explorer. This .skyl file is the audit log file. If you open it in a text editor like Notepad, you will see XML format text. If you are interested in the format, refer to the audit logging paper (in review at *Bioinformatics*). The audit logging file (like the .sky.view which contains window layout and selection information) can be deleted at any time without damaging the document stored in the .sky file.

# Importing Data Files into Skyline

Your next task is to import the mass spectrometer data files from the SRM runs for this experiment:

- On the **File** menu, choose **Import** and click **Results**.
- Use the **Add single-injection replicates in files** option in the **Import Results** form. (default)
- Click the **OK** button.
- In the **Import Results Files** form, navigate to the AuditLog folder and select all of the 9 raw files.
- Click the **Open** button to choose the files.

After the import completes, Skyline should have created 9 new replicates. The **Audit Log** view should now look like this:


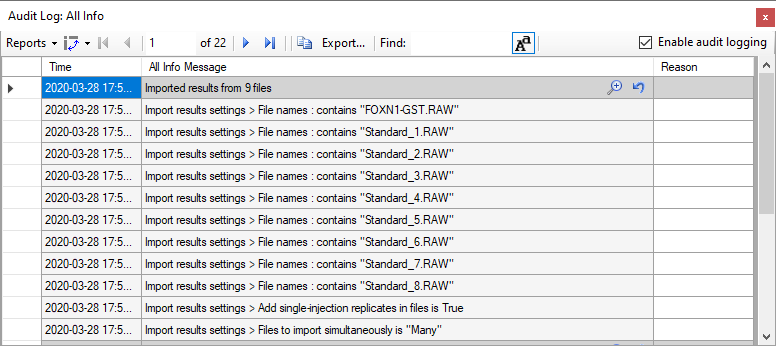


The audit log did not create copies of the files that were imported, but rather just stored the paths of files (here shortened to just the file names). The first 9 detailed messages are the paths of the 9 raw data files you imported. The last two messages describe the settings in the **Import Results** form when you clicked the **OK** button.

Note that the primary entry again has a magnifying glass around a plus sign, which means it has extra information associated with it. This is always the case for a series of dialogs such as when importing results, where you configure a process that modified the document, instead of directly modifying the document like when changing the settings.

- Click on the magnifying glass to view the extra information.


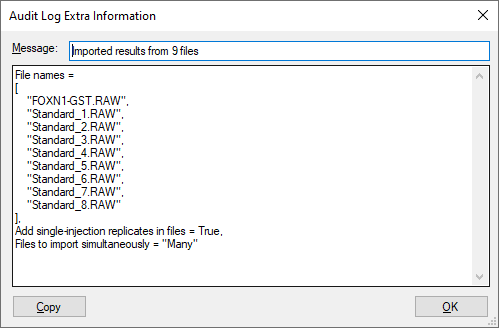


- Click the **OK** button to close the form.

The extra information for wizards like the import results wizard does not contain any additional information, but rather a neatly formatted and more concise version of what is in the audit log. If you have any programming experience, this format might look familiar to you.

# Calibration Curve Settings

Now, configure quantification settings to help Skyline calculate a calibration curve:

- On the **Settings** menu, click **Peptide Settings**.
- Click the **Quantification** tab.
- In the **Regression Fit** dropdown list, choose “Linear”.
- In the **Normalization Method** dropdown list, choose “Ratio to Heavy”.
- In the **Units** field, enter “fmol/ul”.
- Click the **OK** button.

This adds the audit log entry shown below:


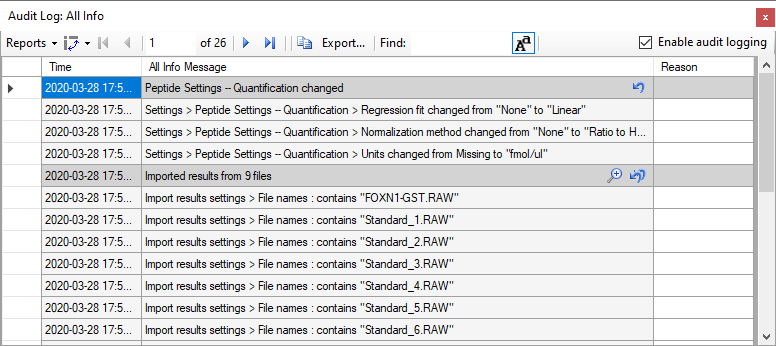


The changes made are listed under the heading **Peptide Settings – Quantification changed** and each step is captured in a detailed message describing it with enough detail that the step could be repeated.

## Specify the analyte concentrations of the external standards:

Next, specify the analyte concentration for each replicate using the **Document Grid** as follows:

- On the **View** menu, click **Document Grid**.
- In the top left of the **Document Grid**, click the **Reports** dropdown list and choose **Replicates**.
- In the row for FOXN1-GST, set its **Sample Type** to “Unknown”.
- Copy the following data and paste it into the **Document Grid** to set each of the “Standard_#” replicates **Sample Type** to “Standard” and their **Analyte Concentrations** to the values below.

| Standard | 40 |
| --- | --- |
| Standard | 12.5 |
| Standard | 5 |
| Standard | 2.5 |
| Standard | 1 |
| Standard | 0.5 |
| Standard | 0.25 |
| Standard | 0.1 |

The **Document Grid: Replicates** form should look like this:


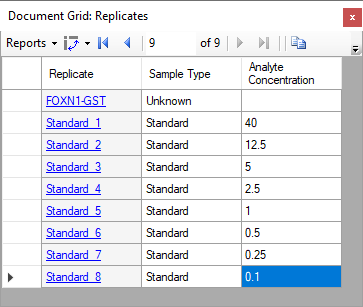


Review the **Audit Log** grid to see that it shows the following:


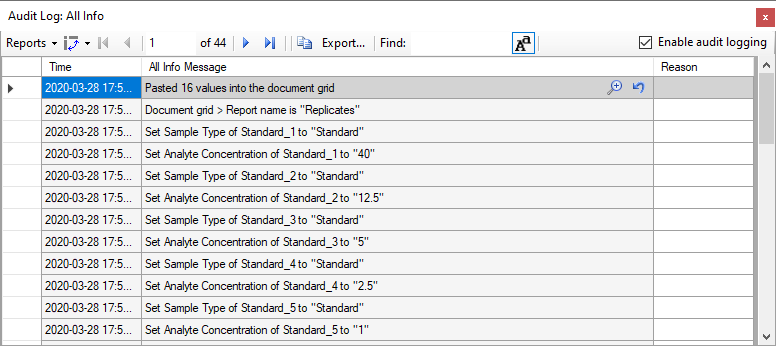


The Undo-Redo messages indicates that 16 values were pasted into the **Document Grid**. There are a total of 17 detailed messages. The first message indicates that the **Replicates** report was used. This is important because in order to reproduce this change made in the **Document Grid**, you need to know what columns were present. The follow 16 messages describe each cell change, one for each of 8 changes in the **Sample Type** column and 8 in the **Analyte Concentration** column. Since you pasted data, this audit log entry again contains extra information.

- Open the extra information window by clicking on the magnifying glass icon.


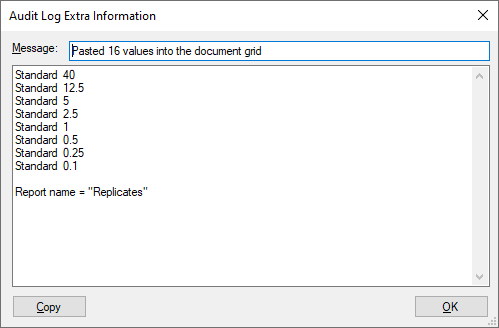


This displays a much more concise view of the data that was pasted into the document and again the data can be easily copied and pasted to reproduce the operation. The report that was used is also indicated at the end of this extra information – in this case: **“Replicates”.**

- Click the **OK** button to close the **Audit Log Extra Information** form.

# Adjusting Integration Boundaries

Next, you will look at the integration boundaries of the peptide in the FOXN1-GST sample:

- On the **View** menu, choose **Arrange** **Graphs** and click **Tabbed**.
- Click on the **FOXN1-GST** tab, or select the **FOXN1-GST** replicate in the dropdown list labeled **Replicates** at the top of the **Targets** view.
- Ensure that the IEAIPQIDK peptide is selected in the **Targets** view.

Now you should see the chromatograms of the IEAIPQIDK peptide in the FOXN1-GST sample.

By selecting either the light or heavy precursor in the **Targets** view you can review the integration boundaries for that precursor.

- Select the heavy precursor and zoom in on the peak using the scroll-wheel beneath the x-axis.

You should see something like the following:


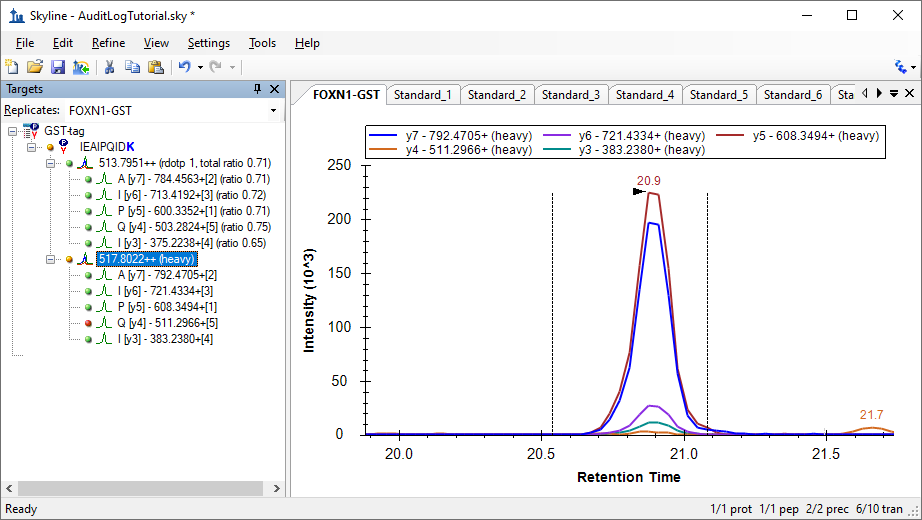


The integration boundaries look acceptable and changing them may simply add variance to the peak area calculations. However, you might feel the peak could be better centered within the integration range. To make a change like this, do the following:

- Click beneath the x-axis where you want the range to start and hold while dragging until the cursor is under where you want the range to end. (e.g. 20.65 to 20.15)

Three new rows should appear in the **Audit Log** view, as shown below:


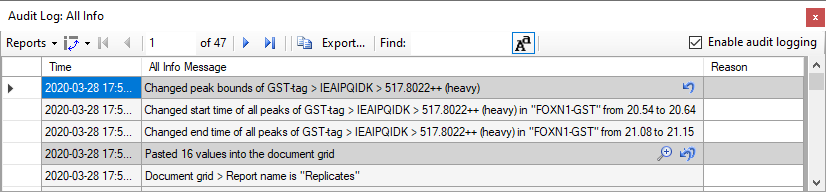


Note that the new log entry describes a change related to an item in the **Targets** window, it uses a format similar to the one seen previously when changing document settings. Generally, the audit log will refer to items in the **Targets** view by **Protein/Peptide List > Peptide > Transition group > Transition**.

When someone else reads this audit log, this integration boundary change might seem arbitrary without any explanation. Or, months or years later, you may have forgotten why you made this change. To document your reasoning do the following:

- Click in the **Reason** cell next to the log message.
- Enter a Reason such as “*Changed peak integration as instructed by the tutorial*” and press Enter.

The audit log should look like this:


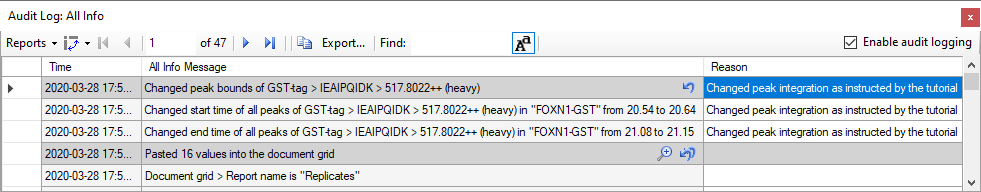


Note that the reason entered in the dark gray row is shown in all three new rows. This is the overall reason for the change. To give a different reason for each row, you would need to use a report field called **Detailed Reason**. You will learn how to customize the columns shown in the **Audit Log** view below.

# Calibration Curve Review

Now view the calibration curve you have configured as follows:

- On the **View** menu, click **Calibration Curve**.

The curve fits the first four points, but not as well for the points closer to zero. Use the scroll wheel to zoom in on the lower four points.


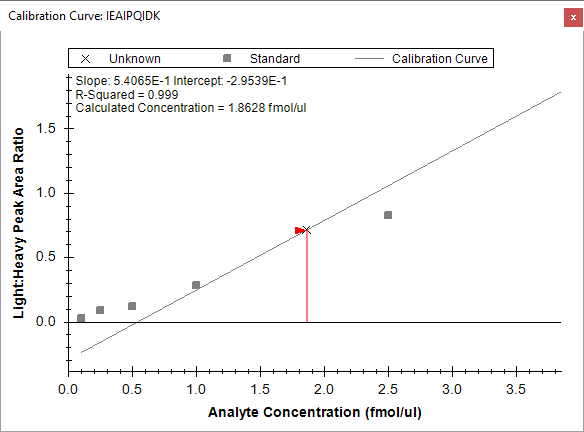


You can see that the last three points fall off the line, which indicates that they are below the limit of detection for this peptide. These three and the fourth lowest concentration point may also be pulling the regression line away from the fourth highest concentration point at 2.5 fmol/ul. To create a better fit for the four highest concentration points, you can exclude the four lower points from the regression as follows:

- For each of the lowest four points, right-click on the point and click **Exclude Standard**.

If you zoom out on the other four points, you should see that the curve fits them better now, and the R-Squared value in the top left corner of the graph should become 1.

Review the **Audit Log** grid and, you should see four new entries, one for each point you excluded from the calibration:


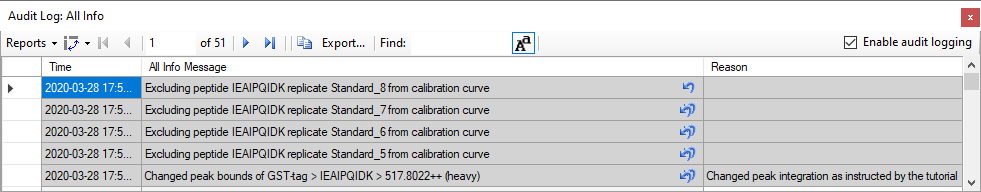


You should again add a reason to explain why the replicates were excluded by doing the following:

- Click on the top **Reason** cell and enter “*Excluded standard below LOD*” and press Enter.
- Select all four **Reason** cells.
- Right-click on the selection, and click **Fill Down**.

# Customizing the Audit Log Display

You now have a growing audit log and it has become difficult to see all your changes at once. The **Audit Log** view is based on the **Document Grid** and can, therefore, be customized just like the **Document Grid**:

- In the top left corner of the **Audit Log,** click on the **Reports** menu.

There are three default reports you can choose from (**Undo Redo**, **Summary**, and **All Info**):


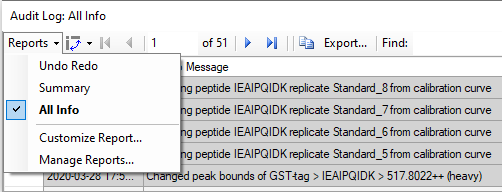


- Click on the **Undo Redo** menu item.
- Adjust the column widths so you can see their full contents.


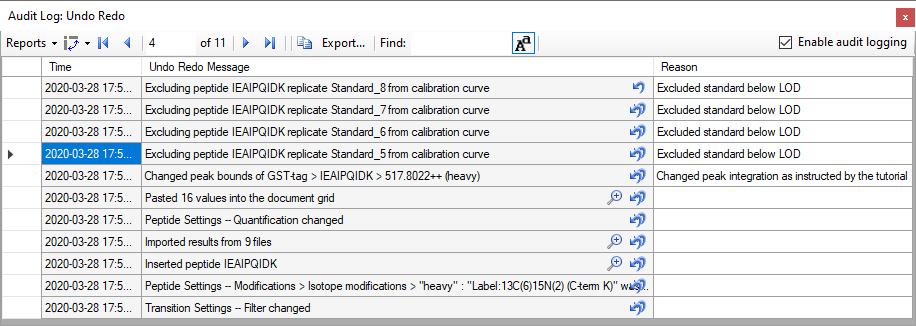


Now the **Audit Log** view only displays the **Undo Redo** messages and not the detail messages, presenting a concise overview of what you changed in the document. Going from bottom to top, your changes can be summarized as follows:

- You first configured the transition settings and peptide settings to include the desired transitions and modifications.
- You inserted the peptide.
- You imported the results.
- You started configuring the calibration curve, by configuring quantification settings.
- You pasted the standard and analyte concentration information into the document grid.
- You adjusted one of the peak boundaries and provided a reason for it.
- Finally, you excluded four standards from the calibration curve, again providing a reason.

Next, you will customize the **Audit Log** view manually to show other columns:

- In the top left corner of the **Audit Log** view, click **Reports** and choose **Customize Report**.
- Check the **Skyline Version** and **User** columns in the left-hand list.
- In the **Report Name** field, enter ‘Custom Columns’.

The filled-out form should look like the following:


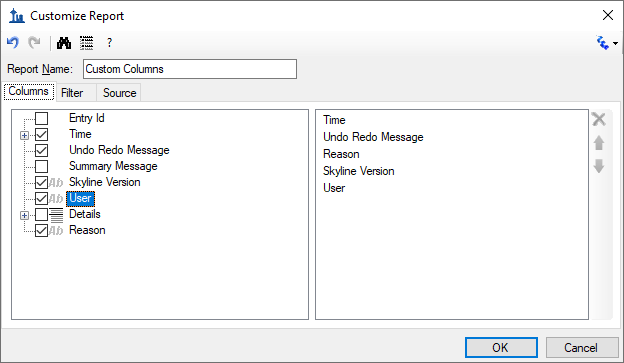


- Click the **OK** button.

The **Audit Log** should now look like the following, although you will have a different **User** name and you might be using a different **Skyline Version**.


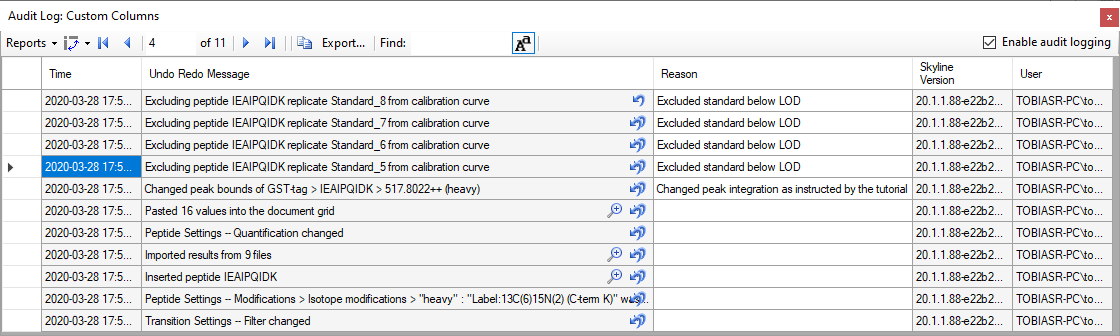


Below is a list of all of the columns you can display and their meaning:

- **Time Stamp:** Time at which the change was made (adjusted to your local time).
- **Undo Redo Message:** The most specific single line message describing the entire document change. This message will also be displayed in Skyline’s toolbar when clicking on the arrows next to the Undo-Redo arrows.
- **Summary Message:** Similar (and often the same) as the Undo Redo Message, but for certain messages shorter.
- **All Info Message(s):** A list of messages that describe the document change in detail.
- **User:** Identity of the user who made the change as authenticated by the local operating system.
- **Reason:** The reason for the change, which can be set by editing the cell in the audit log grid after the change was made. (Optional)
- **Detailed Reason:** A reason that can be set for each of the detailed “All Info messages”. (Optional)
- **Extra Info:** additional information, usually large amount of data pasted into the document. (Optional)

# Panorama Upload

To proceed with the rest of the tutorial you need an account established on PanoramaWeb.org or another web server running Panorama.

Panorama is a web-based proteomics data sharing platform that supports uploading and viewing of Skyline files. To upload your Skyline document into Panorama:

- On the **File** menu, click **Upload to Panorama**.

If you have not used Panorama before the following message should appear:


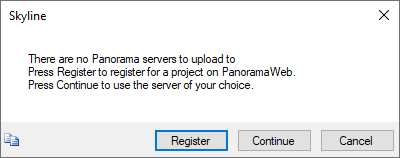


- Click the **Continue** button.

The next form should ask you for the URL of the Panorama server you want to use and your credentials.


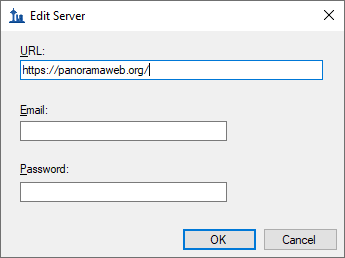


- Enter the URL if you are using your own Panorama server, or leave it as PanoramaWeb.org.
- In the **Email** and **Password** fields, enter your credentials.
- Click the **OK** button.

If the connection was successful, the next form should show you the Panorama server URL and the folders on the server available for upload.


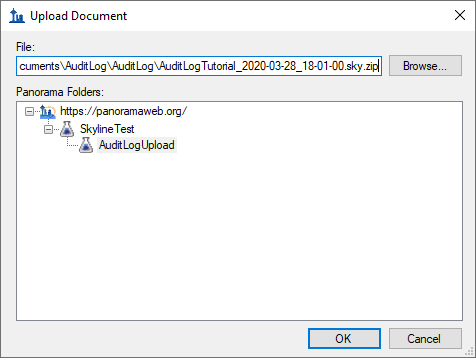


- Click the **+** next to the URL to expand the list of folders you have access to if necessary.
- Select the folder into which you want to upload the document.
- Click the **OK** button.
- Click the **Yes** button in the next form to open a web browser to uploaded document.

Skyline automatically creates a ZIP file containing all the files associated with your document and uploads it to the Panorama server. Click OK when asked to open the document in Panorama. You might be asked to log in to Panorama in the browser first. Once you have signed in the page should look like this:


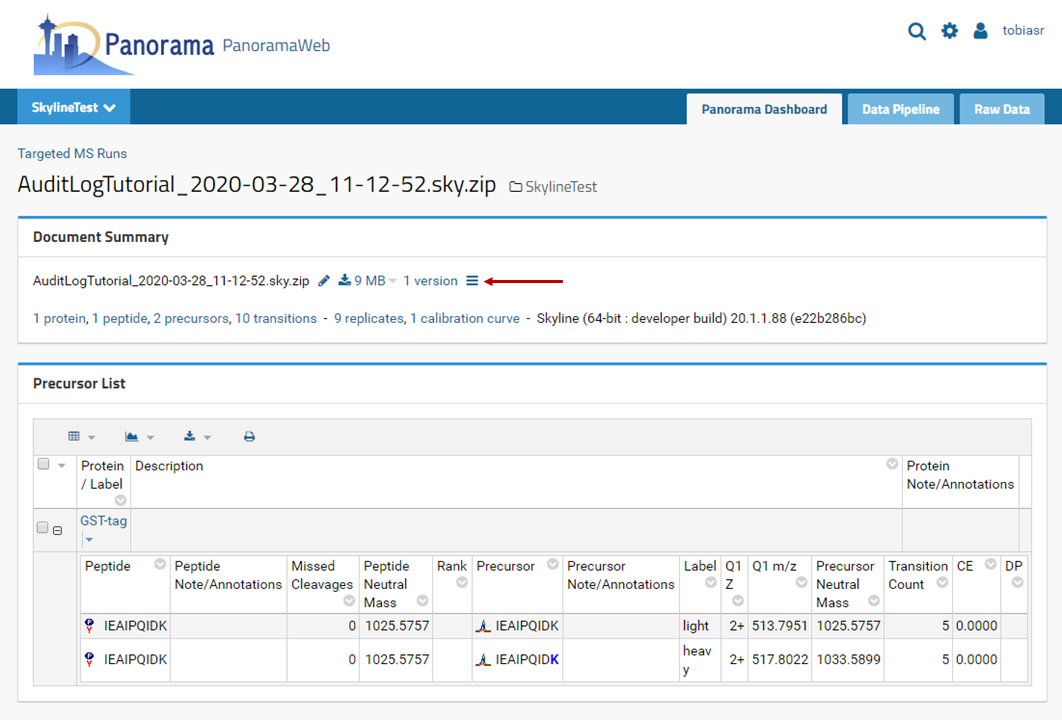


| Detailed discussion of the Panorama functionality is beyond the scope of this tutorial. Please refer to <https://panoramaweb.org/sharing_documents.url> for more details. |
| --- |

Note the ≡ symbol next to the number of document versions. This is a link that allows you to access the audit log information for the document. If you upload a document without a valid audit log this symbol will not be shown.

- Hover mouse cursor over the ≡ symbol to observe the explanatory tooltip.
- Click the ≡ symbol.

Now you should see the following page:


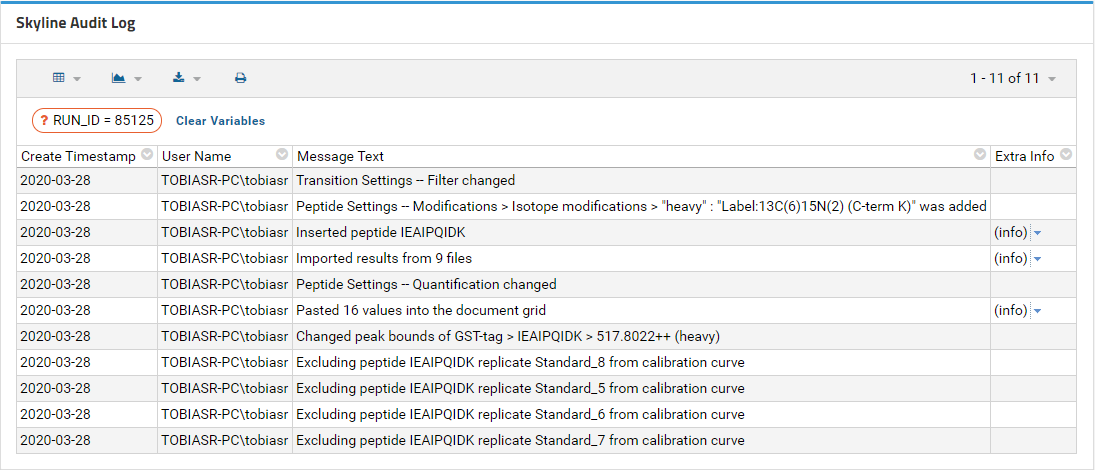


This is an audit log viewer very similar to the Audit Log grid in Skyline. For the sake of brevity, it shows Undo Redo messages only by default, but full details are available through the grid button on the left. It is very similar to the Skyline **Reports** menu and allows you to select, create, and customize grid views. By default, Panorama ships with the **default** view and **AllMessagesView**, but you can also create your own.

- Click on the **Grid View** button
- Select **AllMessagesView** from the dropdown menu.


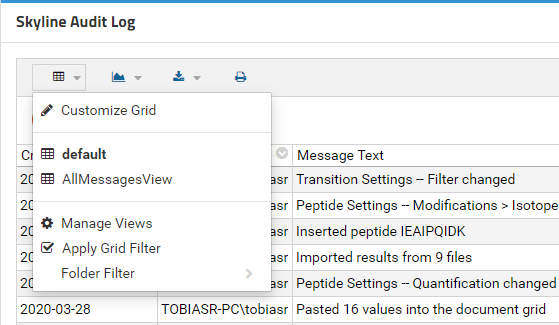


Now your page should look like this:


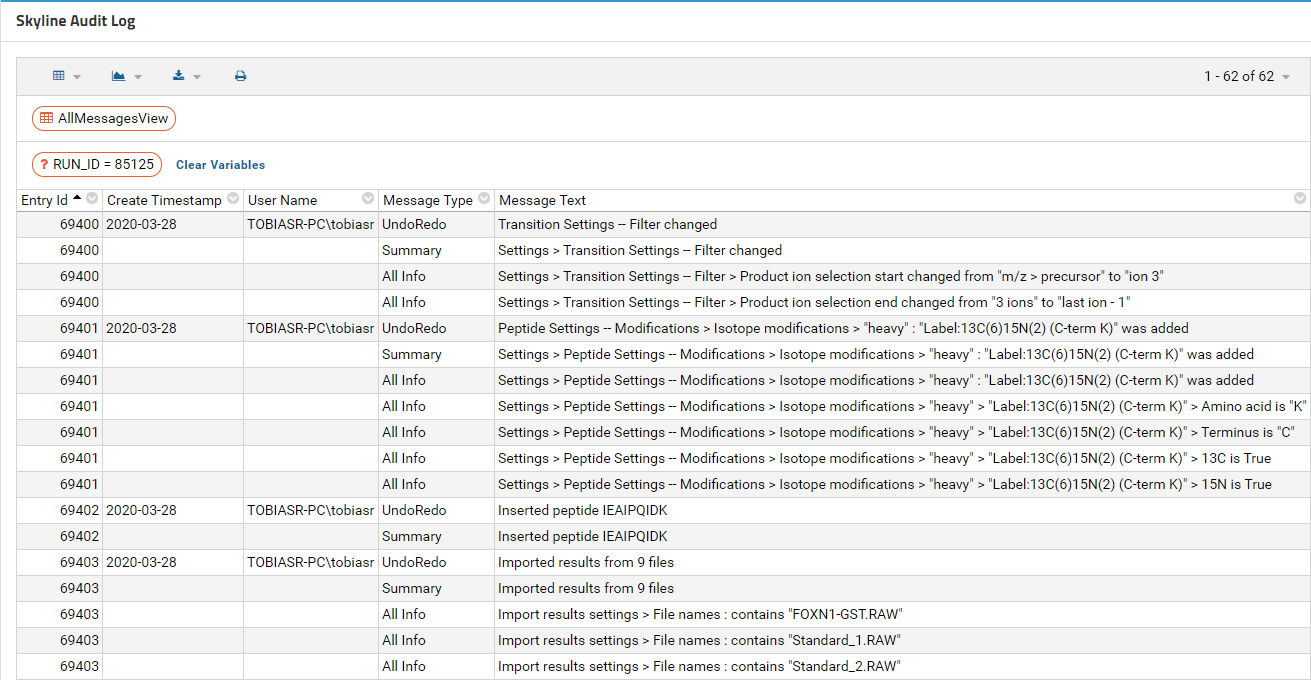


All the messages and message types in the audit log are now displayed.

# Conclusion

This tutorial went through setting up an absolute quantification experiment to demonstrate how to use the Skyline audit log and how to upload it to Panorama. The audit log keeps tracks of all changes you make to your document and is fully customizable. It is a powerful tool to reproduce the state of a document from scratch and can be useful when working on the same document with collaborators or when requesting troubleshooting help from the Skyline team.
